# The Emerging Role of Omics-Based Approaches in Plant Virology

**DOI:** 10.3390/v17070986

**Published:** 2025-07-15

**Authors:** Viktoriya Samarskaya, Nadezhda Spechenkova, Natalia O. Kalinina, Andrew J. Love, Michael Taliansky

**Affiliations:** 1 Shemyakin-Ovchinnikov Institute of Bioorganic Chemistry of the Russian Academy of Sciences, Moscow 117997, Russia; v.sam@ibch.ru (V.S.); solanum@ibch.ru (N.S.); kalinina@belozersky.msu.ru (N.O.K.); 2 Belozersky Institute of Physico-Chemical Biology, Lomonosov Moscow State University, Moscow 119234, Russia; 3 The James Hutton Institute, Invergowrie, Dundee DD2 5DA, UK; andrew.love@hutton.ac.uk

**Keywords:** plant virus, resistance, genomics, proteomics, transcriptomics, metabolomics, multiomics, single cell omics, spatial omics, phenomics

## Abstract

Virus infections in plants are a major threat to crop production and sustainable agriculture, which results in significant yield losses globally. The past decade has seen the development and deployment of sophisticated high-throughput omics technologies including genomics, transcriptomics, proteomics, and metabolomics, in order to try to understand the mechanisms underlying plant–virus interactions and implement strategies to ameliorate crop losses. In this review, we discuss the current state-of-the-art applications of such key omics techniques, their challenges, future, and combinatorial use (e.g., single cell and spatial omics coupled with super-resolution high-throughput imaging methods and artificial intelligence-based predictive models) to obtain new mechanistic insights into plant–virus interactions, which could be exploited for more effective plant disease management and monitoring.

## 1. Introduction

### 1.1. Plant–Virus Interactions

Plant viruses are a major threat to crop production that could result in significant yield losses of up to USD 30 billion worldwide per annum [1,2]. To mitigate this, we need to better understand the underlying molecular mechanisms underpinning crop plant invasion and development of symptoms in order to identify key disease regulators which may be targeted to block these processes. This complex and time-consuming task has been made easier with current advances in omics technologies.

In plants, viruses orchestrate a large range of multifarious genetic, metabolic, and physiological changes, within which a suite of these responses may promote virus replication and host susceptibility [3,4]. These initial changes are responsible for establishing successful invasion of the crop plants and often involve the virus switching off effective plant defences and deploying counter defence strategies to inhibit host responses which may block virus ingression. In order to attempt to protect themselves from virus infections, plants have evolved several resistance mechanisms that act in a complementary manner, such as PAMP (pathogen-associated molecular pattern) triggered immunity (PTI) and effector-triggered immunity (ETI) [5]. PTI is a very early broad-spectrum defence response which occurs when host plant hypothetical pattern recognition receptors (PRRs) initially detect viruses via their PAMPs (such as double-stranded RNA (dsRNA) replication forms) [6], triggering a cascade of intracellular defensive signalling events, including the generation of reactive oxygen species (ROS), increased production of defence hormones (such as salicylic acid (SA), and activation of mitogen-activated protein kinases 3 and 6 (MAPK3/MAPK6)) [7]. In addition to PTI, some viral proteins (which act as effectors) may interact with specific intracellular receptors known as R gene proteins which can subsequently induce a more intensive and robust antiviral defence (ETI) [5]. Functional R proteins can be composed of three domains: a central nucleotide-binding site (NBS) domain, a leucine-rich repeat (LRR) domain, and a N terminal Toll Interleukin-1 receptor (TIR) or a coiled-coil (CC) domain. The combined action of PTI and ETI may induce systemic acquired resistance (SAR), which is characterised by the development of resistance in tissues distal to the infection site, with SA being the principal plant hormone responsible for establishing SAR [8]. When the host plant successfully deploys these strategies, increases in ROS may directly attack the pathogen and when coupled with SA induction, this may lead to downstream tissue necrosis and callose deposition which may serve to localise the virus at the point of entry, thus limiting virus invasion.

Gene silencing or RNA interference (RNAi) is another major mechanism which can defend plants from viral attack. This is a nucleotide sequence-specific mechanism that involves production of small RNAs which activate the silencing machinery to target complementary DNA or RNA for transcriptional (TGS; i.e., nucleic acid methylation) or post-transcriptional (PTGS; i.e., degradation of specific RNAs or their translational repression) silencing, respectively [9,10]. In the context of RNA viruses, dsRNA molecules formed during virus replication are recognised and cleaved by Dicer-like proteins (DCL) into small interfering RNAs (siRNAs). Consequently, these siRNAs are loaded into ARGONAUTE (AGO) protein family member structures to form an activated RNA-induced silencing complex (RISC) which operates to specifically degrade complementary viral RNAs [9,10,11,12]. Host RNA-dependent RNA polymerases (RDRs) such as RDR6 also play an important role in antiviral RNA silencing by amplifying the synthesis and spread of virus-specific siRNA molecules in planta [13].

Plant viruses are obligate intracellular parasites and, due to their small genomes, are reliant on hijacking or subverting host proteins and cellular structures and functions for successful replication and infection. These include eukaryotic translation initiation factors [14], heat shock protein 70-2 (Hsc70-2) [15], subnuclear proteins, fibrillarin [16,17], and coilin [17,18], and many others. These reports demonstrate how plant viruses can divert cellular components from their original roles to pro-viral functions. The analysis of compartments that lack surrounding membranes, but concentrate biomolecules including proteins and nucleic acids (termed biomolecular condensates in plants), clearly showed that plant viruses can enhance their invasion by recruiting hundreds if not thousands of pro-viral cellular components into viral condensates to provide a favourable environment for virus accumulation [19]. Viruses can also completely reprogram cellular regulatory systems for successful infection, such as posttranslational protein modifications for the orchestrated regulation of viral and cellular protein dynamics, and they can subvert glycolysis and fermentation pathways to use host resources. They can also hijack amino acid and secondary metabolite biosynthesis, including flavonoid and phenylpropanoid pathways, thereby weakening plant defences and modulating ion homeostasis to create a cellular environment that is favourable for viral genome replication [20]. Recent advancements in genome technologies, such as CRISPR/Cas9 or RNA interference offer innovative strategies to mitigate all these impacts. Precise genetic modifications can restore or optimise disrupted signalling and metabolic pathways to pave a way for development of novel antiviral strategies.

An important step in this direction is to identify potential gene targets for gene editing or RNAi approaches. The concept of omics-based technologies facilitates a deeper understanding of the mechanisms underpinning plant viral diseases and hence provides important information on host genes involved in plant–virus interactions and disease control.

### 1.2. Introduction to Omics: Basics

**Figure 1:**
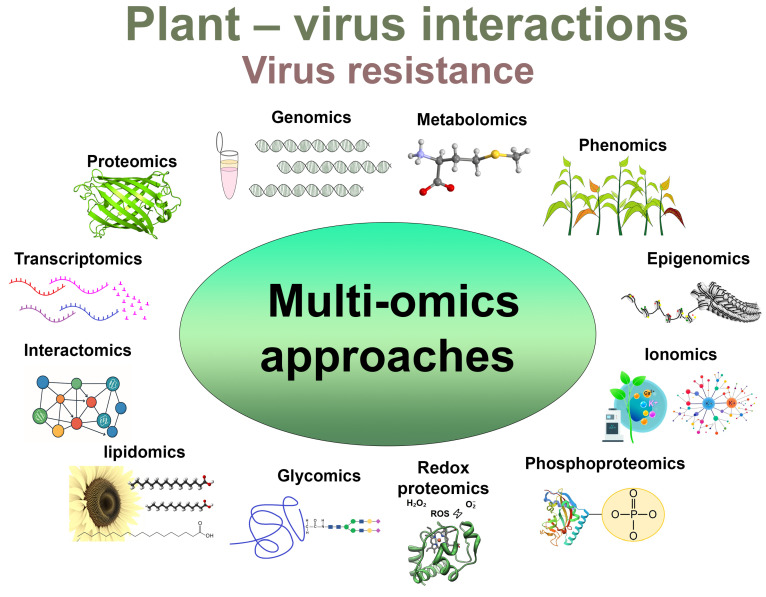
The role of diverse omics technologies including genomics, transcriptomics, proteomics, metabolomics, epigenomics, phenomics, and multi-omics in understanding the plant–virus interactions and mechanisms underlying virus resistance. Genomics deciphers an organism’s entire DNA genomic sequence. Transcriptomics provides information into how genes are expressed via transcription of RNA. Proteomics identifies whole sets of protein molecules which are produced in cells under different conditions. Metabolomics focuses on the analysis of a set of metabolites, consisting of small molecules produced by cells that are responsible for biochemical processes in organisms. Epigenomics analyses changes occurring with chromosomes may modulate the expression of a gene assumed to be active from the genomics study. Phenomics aims at collecting non-invasive imaging (i.e., RGB, chlorophyll fluorescence, hyperspectral and thermal imaging, and spectroscopy data) and developing comprehensive tools to integrate with other omics data. Ionomics measures the complete set of ions. Phosphoproteomics is a branch of proteomics that describes proteins containing a phosphate group as a posttranslational modification. Redox proteomics focuses on oxidatively modified proteins. Glycomics identifies the structure and function of the complete set of glycans (the glycome). Lipidomics analyses cellular lipids. Interactomics studies interactions between biomolecules, particularly proteins. Multi-omics (also known as integrative omics) analyses and integrates data from multiple omics—genomics, transcriptomics, proteomics, metabolomics, and others.

The term “omics” is derived from the Greek word “ome” (-ōma), which means “whole”, and when it used as a suffix refers to collective and universal characterisation and quantification of entire sets of different types of biomolecules such as genes (genomics), proteins (proteomics), RNA transcripts (transcriptomics), or metabolites (metabolomics) in a specific biological sample (Figure 1).

Genomics [21] deciphers an organism’s entire DNA genomic sequence, identifies genes and explores their structure and functions. Recent progress in the development of advanced next generation sequencing (NGS) technologies, such as Illumina, PacBio, and Oxford Nanopore has revolutionised the whole area of genomics; enabling a fast, cost-effective, and high-throughput analysis of complete genomes. These advances have led to real breakthroughs in fields like medicine and agriculture.

Transcriptomics [22,23] provides key insights into how genes are expressed via the transcription of RNA, with RNA sequencing (RNAseq) technologies permitting analysis of the dynamics of gene activities involved in critical cellular responses.

Proteomics [24,25,26] identifies whole sets of protein molecules which are produced in cells under different conditions. Modern proteomics often uses mass spectrometry (MS) approaches, as a driving force to analyse the composition of proteins, their isoforms, their structure and modification status. Proteomics is a powerful tool for generating a map of the functional pathways, networks, and molecular systems which control the major cellular responses, particularly when combined with complementary data derived from potent genomics and transcriptomics methodologies.

Metabolomics [25,26] is the study of the metabolome, which is a set of metabolites, consisting of small molecules produced by cells that are responsible for biochemical processes in organisms. Metabolomics, unlike genomics, transcriptomics, and proteomics, provides a real-time snapshot of an organism’s physiological state which is directly correlated with phenotype; for instance, changes in metabolic profiles can be an early marker of disease. Nuclear magnetic resonance (NMR) spectroscopy and high-resolution MS have become cornerstones of metabolomic research.

In addition to the omics strategies discussed above, an increasing number of studies also incorporate relatively newer omics approaches including epigenomics, interactomics, lipidomics, ionomics, microbiomics, glycomics, redox proteomics, and phosphoproteomics (Figure 1) [25] which would open up new opportunities in analysis of plant–virus interactions. Furthermore, other omics complementary to genomics are emerging, such as phenomics with its emphasis on high-throughput phenotyping using imaging technologies to correlate phenotypic changes with molecular data [25]. With a prime focus on the “four big omics”—genomics, transcriptomics, proteomics, metabolomics—we will outline their links with other omic technologies, in particular, with regard to future plant virus research. This review will mainly contemplate RNA virus–plant interactions particularly providing some examples from our own research carried out on plants of the family *Solanaceae*. This family is considered one of the most important families among plants because it includes many staple food crops and species rich in essential secondary metabolites [27]. However, omics technologies have also been successfully applied to many other crops and species which is illustrated in Table 1. Moreover, information in this table is not restricted by RNA viruses and includes various DNA viruses, which, however, may require a modified analytic strategy especially in the genomic and transcriptomic platforms.

## 2. Application of Genomics in Plant Virus Disease Control

In crop agriculture, genomic studies focus on the total number of genes, their structure, organisation, mapping and functional roles in different biological processes and their annotation [21]. There are two broad categories of studies that are key in the modern genomic field. One of them, namely structural genomics, deals with sequencing, assigning, and mapping the genes and markers onto the chromosomes, thereby constructing a physical map of the whole genome. Advances in the development of next generation sequencing technologies, such as Illumina (for short reads), PacBio, and Oxford nanopore (for long reads), have improved the genomic studies through faster, cheaper and more accurate sequencing, which is further enhanced when used in conjunction with current bioinformatic data analysis. Thus, given that plant genes and therefore genotype control the final physiological profile (phenotype), structural genomics appears to be an entry point and fundamental basis for other ‘omics’ approaches. Genomic studies have also enabled the detection of single nucleotide polymorphisms (SNPs) or simple sequence repeats (SSRs), which may serve as molecular markers located throughout the genome of agronomically important crops [83,84,85]. Such molecular markers exhibit significantly enhanced density and higher resolution compared with quantitative trait loci (QTL) [86]. These markers may be exploited in genome-wide association studies (GWAS), which have become a powerful tool in genomic-assisted breeding methodologies, analysing statistically significant associations between a phenotype/trait (such as virus resistance) and genetic variants in large collections of germplasm [87]. GWAS identify potential trait-associated markers (typically SNPs) by measuring the frequency of SNP sites in samples with or without a specific trait (virus resistance), thereby aiming to identify genetic variations that are significantly enriched in samples with the trait of interest.

The other category of genomics, namely functional genomics, integrates nucleotide (genomic) sequences with gene functions at the whole genome level via using high-throughput functional genomic screens such as genome-wide knockout/knockdown screens obtained by mutagenesis, RNAi or CRISPR-Cas technologies [88]. Agronomically important genes may be thus identified and targeted for trait improvement including virus resistance.

With both these strategies (GWAS and functional genomics), genomic regions/candidate gene(s) controlling viral disease resistance have been identified in more than 15 plant species and crops. GWAS has been used to map resistance against more than 25 RNA-containing and DNA-containing viruses including such economically important viruses as potato virus Y (PVY) [89], cucumber mosaic virus [90], rice black-streaked dwarf virus [91], cassava mosaic virus [92], tomato spotted wilt virus [93], and some others [84,87,94]. Functional genomics approaches has allowed the identification of a broad number of plant host gene-determinants of susceptibility to various viruses [95], which is an important step for engineering virus resistant plants using CRISPR-Cas editing or RNAi technology.

Furthermore, another area related to genomics is comparative population genomics, which involves comparing genomic features across different species and among different populations with a focus on genes, gene domains, structural motifs, regulatory sequences, and other genomic signatures to explain evolution, structure, and function [96]. With regard to plant viral diseases, population genomics studies viral genotype—disease phenotype relationships, genetic diversity, and evolution of viral populations, emergence of new viruses and their strains/isolates, and environmental and geographical heterogeneity of viral populations [97]. It is worth noting that for RNA-containing viruses which represent the vast majority of plant viruses, genomic studies are based on RNA sequencing (RNA seq; see section below). On a practical level, this information is very important due to the necessity to identify conserved virus genetic regions which may be broad-spectrum targets for viral disease control using CRISPR-Cas or RNAi approaches.

## 3. Application of Transcriptomics in Plant Virus Disease Control

RNA molecules are an important component of organisms. Understanding the identity and abundance of each RNA species in a specific cell under a certain condition is crucial for RNA research [98]. Transcriptomic studies allow for the identification and quantification of the majority of RNA transcript species: mRNA, long non-coding RNAs (lncRNAs), and small RNAs (smRNAs) [22]. These can be rapidly and accurately analysed robustly using high-throughput RNA next generation sequencing (RNAseq) such as Illumina or Oxford Nanopore platforms [98].

Transcript identification and quantification are the primary goals of the RNAseq analysis [99], which is often followed by workflows which determine the biological relevance of the data. Using differential expression analysis (which may also include assessment of differential isoform expression and alternative splicing) and functional profiling can help elucidate the molecular mechanisms and key pathways controlled by differentially expressed genes (DEGs) under the experimental/field test conditions [100]. For RNA-Seq data, quality control is first performed using FastQC or MultiQC tools [101]. Then, low-quality reads are removed using Cutadapt [102] or Trimmomatic [103]. Reads are then aligned to the reference genome using STAR or HISAT2 [104,105], after which the number of reads per gene is counted (featureCounts, HTSeq). Since RNA-Seq data are depth-dependent, they need to be normalised, which often requires methods such as Fragments Per Kilobase Million (FPKM) or Reads Per Kilobase Million (RPKM); however, Transcripts Per Kilobase Million (TPM) is now becoming more popular. Once DEGs have been identified, it is important to understand their biological roles. GO (Gene Ontology) analysis helps to determine the biological processes, cellular components, and molecular functions to which these genes are related, while KEGG (Kyoto Encyclopedia of Genes and Genomes) and Reactome provide information on a wide range of pathways, including signalling, metabolism, and genetic information processing. Tools like DAVID, g:Profiler, and clusterProfiler can automate the identification of enriched GO terms. For effective interpretation and presentation, the results of differential gene expression analysis can be visualised using heatmaps or volcano plots. Subsequently, interaction networks (STRING, Cytoscape) can be established to help to identify the key regulatory genes, which can be confirmed using qRT-PCR to check their expression. This data may also be integrated with proteomic or metabolomic data in order to help establish biological interpretation; a detail oriented process which may be simplified using contemporary bioinformatics tools [100].

Bioinformatic analysis of RNA-containing virus populations in plants is a complex multi-stage process that includes data collection, processing, interpretation, and visualisations. Such analysis allows studying the genetic diversity of viruses, their evolution, interaction with host plants, and pathogenicity mechanisms. After receiving raw data from the sequencer, quality control is performed using FastQC or MultiQC tools [101]. Adapters, low-quality reads, and host plant sequences are then removed (Bowtie2, BWA, or Kraken2 are used). De novo methods (SPAdes, Trinity) or alignment to reference genomes (BWA, minimap2, Hisat2) are used to assemble viral genomes, which may be identified using database searches (BLAST, VirusDetect) [106,107,108,109]. Concomitantly, the genetic diversity and structure of populations may be studied by considering the SNPs, indels and recombinations (GATK) that may be present in the datasets, with the RDP4 programme being used to identify recombinant strains. Evolutionary relationships between strains may be assessed using phylogenetic analysis (MEGA, RAxML, UGENE); however, more in-depth analysis of quasi-species, which are closely related variants within one host, may be further characterised using ShoRAH and Haploflow [110,111,112,113,114,115]. After genome assembly, open reading frames (ORF) are annotated using Prokka or ORFfinder tools and the resulting sequences are compared with databases (NCBI, UniProt) to determine gene functions [116,117,118,119]. Of particular interest are genes associated with virulence as well as the prediction of virus–plant interactions which may be analysed, respectively, using VFDB/PHI-base and KEGG. If the sample contains multiple different viruses or microorganisms, taxonomic classification may be required which would utilise Kraken2 pipeline [120,121,122]. For visual presentation of data, genomic browsers (IGV, Tablet), circular diagrams of genomes (Circos), phylogenetic trees (FigTree, IQ-Tree), and heatmap analysis (Matplotlib, Seaborn) are used, often in conjunction with statistical processing which is performed using R or Python v3.11.3 [123,124,125,126,127].

Transcriptomic studies have provided significant insights in the understanding of numerous plant–virus interactions in model and crop plants. Viruses induce tremendous transcriptomic changes in infected plants which have been recently comprehensively reviewed by Rurek and Smolibowski [23]. These changes are typically associated with carbohydrate transport, cell cycle regulation, DNA binding, DNA repair and recombination, DNA replication, glutathione binding, lipid metabolism, microtubule-based movement, oxidation-reduction proteins, OXPHOS activity, programmed cell death regulation, protein domain specific binding, protein folding and processing, regulation of hydrogen peroxide metabolism, and senescence as well as signal transduction (including hormone signalling in host antiviral defences).

It should also be noted that the advent of RNAseq methodology has allowed the discovery of an entire new category of plant non-coding RNAs involved in viral infections including various smRNAs, miRNAs, and lncRNAs [22]. Transcriptomic profiling has revealed novel associations of NBS-LRR resistance genes with virus-derived or induced products in both compatible and incompatible plant virus interactions [42,128,129]. Regarding the virus input, the discovery of viral small interfering RNAs (vsiRNAs) indicates the potential of transcriptomic approaches to uncover novel mechanisms underlying plant defence responses [130]. We have found that the external application of PVY-specific double-stranded RNA (dsRNA) triggers RNAi-based defence responses, which surprisingly led to formation of a non-canonical pool of smRNAs, which were present as ladders of ~18–30 nt in length (instead of typical virus induced discrete 21 and 22 nt siRNA species); suggestive of a new unexpected pathway of smRNA biogenesis [131].

We have also studied the effect of enhanced temperature on the transcriptomic responses of potato plants to infection with PVY which significantly increased plant susceptibility to this virus. Using RNAseq technology, we found that both single and combined PVY and heat-stress treatments caused dramatic changes in gene expression, affecting the production of both protein-coding and non-coding RNAs [47]. By analysing expression of the newly identified genes responsive to PVY infection, we found that the increased susceptibility of potato to PVY at higher temperature may be caused by the down-regulation of methionine cycle (MTC) enzymes [59], which disturbs the MTC and its major functions in trans-methylation and polyamine production. Other transcriptomic changes revealed in this work were associated with the process of poly ADP-ribosylation (PARylation), suggesting that PVY–plant interactions are at least partially regulated by PARylation functions. We also identified a range of novel non-coding RNAs which were differentially produced in response to single or combined PVY and heat stress, which consisted of antisense RNAs and RNAs with miRNA binding sites. Finally, we identified several sites for alternative splicing and RNA methylation during combined stress conditions. These findings provide insights for future studies of functional links between PVY infections and transcriptome reprogramming, RNA methylation, and alternative splicing.

Another transcriptomic study has demonstrated that Zn^2+^-mediated antiviral resistance in *N. benthamiana* is activated by ethylene responsive transcription factors which enhances the expression of resistance-related genes [132].

Transcriptomic technologies developed to study the RNA transcripts (RNAseq analysis) of an organism have also been successfully employed over the last decade for the detection and discovery of new and emerging plant viruses, which are often associated with diseases in a wide range of host crops such as apple, citrus, nectarine, grapevine, raspberry, blackberry, rose, carrot, eggplant, potato, and others [97,133,134,135].

## 4. Application of Proteomics in Plant Virus Disease Control

Proteomics plays a significant role in elucidating key biological mechanisms such as growth and differentiation, responses to biotic and abiotic stresses, programmed cell death, etc. With rapid advances in the development of mass spectrometry (MS) techniques and computational improvements, during the past decade, MS-based proteomics has emerged as a powerful tool for investigating protein structure, functions, and global interactions in different areas of life sciences and has become increasingly important in plant and crop sciences. The quantitative, functional, spatial, and temporal diversity of plant proteins is controlled by multiple environmental factors that modify protein structure, abundance, their interaction patterns in order to meet the dynamic demands of plants.

Quantitative proteomics is a crucial technique for measuring differences in protein accumulation and post-translational modification rates [136,137]. The major types of genome-wide quantitative proteomics typically applied in plants include stable isotope labelled amino acid methods such as Tandem Mass Tag (TMT), Isobaric Tags for Relative and Absolute Quantitation (iTRAQ), and Stable Isotope Labelling by amino acids in Cell culture (SILAC) [138,139,140]. The latter of these pipelines operates in vivo and incorporates stable isotopes into protein molecules during their synthesis in growing cells or tissues [140]. The labelled proteins/peptides are consequently examined by tandem MS to obtain relative differences in multiplexed samples [141]; a highly efficient process for analysing low-abundance proteins.

Bioinformatics methods for MS-based proteomics data analysis have recently been reviewed by Chen et al. [142], and for more information we would like to refer the reader to this article. In brief, the data from the MS analysis are used to determine the sequence of the peptides via database search algorithms such as Mascot [143] or SEQUEST [144] or de novo peptide sequencing [142]. The next steps include the reconstitution of the peptide sequences into proteins (if possible) using, for example, Bayesian Inference Models [145] and the subsequent determination of protein abundance after normalisation (for instance using Peaks Studio Quantification [142], if number of identified peptides is low). To identify the biological pathways and processes that are associated with over- (or under-) represented proteins, KEGG and GO protein enrichment tools are usually employed [142] as described for transcriptomic analysis.

Post-translational protein modifications play important regulatory roles in protein functions by changing their activity, localization, turnover, and interactions with other molecules and include phosphorylation, ubiquitination, SMALL UBIQUITIN-LIKE MODIFIER (SUMO)-lyation, acylation, methylation, and glycosylation [136,146,147]. Some such modifications can be accurately detected and quantified using high-throughput proteomics [148].

Numerous reports have described various approaches using proteomics to elucidate plant responses to various abiotic and biotic stresses, including virus infections. There is a growing body of information showing the tremendous effects of viruses on the accumulation of proteins involved in core plant metabolism, including photosynthesis and carbon and amino acid metabolism, light harvesting, photorespiratory metabolism, ribosome maintenance and function, splicing, plant immunity, translation, and protein processing and protein degradation [24,70,71,149,150,151,152]. It cannot be dismissed that some of these effects may be caused merely by general stress responses during infection. However, taking into account that many viruses have been shown to interact directly or indirectly with metabolic proteins, it is plausible that this regulation has specifically evolved due to benefits obtained either by the virus during invasion or by the plant host during the defence response.

Unlike a genome which remains relatively static, a proteome is highly dynamic and changes in response to environment factors or disease, developmental stages, and plant age.

Differential regulation of protein accumulation during viral infection is a complex process which depends on a virus species and virus isolate, host species, infection stage, plant age, and environmental conditions. How these circumstances complicate interpretation of the results compounded with complexities of comparing the data obtained in different laboratories will be discussed below. However, here we provide an example illustrating how enhanced temperature may affect protein composition in virus-infected plants. It is well-known that plant–virus interactions are greatly influenced by various ecological factors including temperatures [151,153,154]: increased temperature is often associated with more severe symptoms and higher virus accumulation. We found that infection with PVY at 28 °C (which significantly facilitates susceptibility to the virus) induced much greater changes in the abundance of proteins (152 proteins) than at 22 °C (23 proteins) [59]. A striking finding, for example, was that the enzymes of the methionine cycle (MTC) were down-regulated at the elevated but not at the normal temperature. Consistently, we found that higher temperature conditions induced changes in the level of MTC metabolites, suggesting that the enhanced susceptibility of potato plants to PVY at 28 °C may be caused by the down-regulation of MTC enzymes and concomitant cycle perturbation. In agreement with this, exogeneous treatment of these plants with methionine restored accumulation of MTC metabolites and subverted the susceptibility to PVY at higher temperature [59].

## 5. Application of Metabolomics in Plant Virus Disease Control

Recent advances in the development of metabolomics, particularly high-resolution platforms such as liquid chromatography–mass spectrometry (LC-MS) and gas chromatography–mass spectrometry (GC-MS) as well as tools such as MetaboAnalyst and XCMS used for high-throughput data processing [20,155,156] have allowed researchers to identify many dynamic metabolic changes in plants under stress conditions including virus infections. Viruses are able to significantly affect the plant metabolism, including primary metabolic pathways which are closely related to energy production and plant growth as well as secondary metabolic pathways which are crucial in abiotic and biotic stress responses. For instance, metabolomic studies showed that tomato yellow leaf curl virus causes remarkable up-regulation of the phenylpropanoid and ureide/polyamine pathways, resulting in the accumulation of flavonoids and lignin, thereby increasing antiviral resistance [157]. Similarly, horse gram yellow mosaic virus induced different metabolite changes in highly resistant and highly sensitive genotypes of horse gram. Significantly enhanced production of sugars (which may act as signalling molecules in plant–pathogen interactions), alkanes (derivatives of cuticular wax providing tolerance against biotic stress), carboxylic acids (sugar signalling pathway which may be related to systemic spread of the virus), and three antiviral compounds (octadecanoic acid, diphenylsulfone, and 2-aminooxazole) was observed in the highly resistant genotype of horse gram [68]. Investigations on the Sri Lankan cassava mosaic virus demonstrated the central role of chlorogenic acid and caffeic acid in ROS regulation and defence pathways in cassava plants [81].

## 6. Challenges and Pitfalls of Individual Omics Technologies

Recent advances in high-throughput omics technologies have dramatically changed research in plant sciences and agriculture. In spite of the great potential of these technologies, the implementation of omics still faces serious technical and analytical challenges. These challenges may arise at every stage, from sample preparation and data generation to interpretation of extensive datasets.

With regard to NGS approaches (in genomics and transcriptomics), the presence of highly repetitive DNA in plant genomes as well as heterozygosity and polyploidy of agricultural crops could produce hindrances during assembly of short reads obtained during sequencing using Illumina. Long reads obtained using PacBio or Oxford Nanopore may contain significant inaccuracies, which may be eliminated, at least partially, by error self-correction tools such as DeChat [158]. Bioinformatic analysis is another bottleneck. Transcriptome assembly is particularly challenging for alternative splice variants, novel genes, and reads that map to multiple regions of the genome (multi-mapping), making quantification of expression difficult. Different normalisation methods (RPKM, TPM, DESeq2) can yield divergent results, and batch effects can mask biologically significant differences [159,160,161,162].

In contrast to the genome, transcriptomes and, specifically, proteomes and metabolomes are highly dynamic networks that continuously change according to physiological and environmental conditions, and are strictly dependent on crop, cultivar, developmental stage, tissue or cell type or cellular compartment [26,149]. If the sample consists of a mixture of cell types, the signal is averaged out, and important changes in individual populations of RNA transcripts/proteins/metabolites may go undetected. In addition, when testing thousands of transcripts/proteins/metabolites en masse, false positives are inevitable, requiring independent validation. Moreover, it is not surprising that homologs of the same transcript or protein may be differentially regulated in opposite directions under certain conditions, e.g., during virus infection, in different plant species, or at different times, and it is not unusual for two homologous transcripts/proteins to be regulated in opposite directions even in the same study and at the same time. This makes it rather difficult to predict the functional role of these proteins in the process under study. It is even more difficult to compare and interpret results obtained by different research groups under different conditions (such as time points, temperatures, plant species and age) [149]. Another concern is that functional identification of differentially expressed transcripts/proteins requires a comprehensive well-annotated database of reference sequences for the plant species of interest. However, for some plant species, such databases appear to be quite poor or not available at all. Regarding proteins, functions are being frequently predicted by homology searches using available databases, which may result in wrong predictions. Proteomics, in contrast to transcriptomics, faces additional challenges due to their post-translational modifications.

Metabolic variability may cause problems in identification of the metabolites associated with a certain trait. Most of the metabolites are typically involved in multiple pathways; this makes it difficult to attribute some metabolites to a specific biochemical pathway. Extraction of metabolites is a critical step where significant losses are possible. The choice of solvent affects the extraction efficiency: polar metabolites (sugars, amino acids) require aqueous solutions, while lipids and hydrophobic compounds are better extracted with organic solvents. However, there is no universal method, which leads to a systematic bias towards certain classes of compounds.

Integration of multi-omics data including genomics, transcriptomics, proteomics, and metabolomics may at least partially overcome these challenges and increase accuracy, reliability and robustness of omics-based insights.

## 7. Multi-Omics

Individually, each omics technology generates quite large datasets; however, this relates solely to a single level of information in a biological system which may introduce some confusion in understanding and prediction of the entire process. Therefore, to avoid misinterpretations and provide comprehensive information on the full flow of genetic information from genotype to phenotype, multi-omics studies are required [163]. A typical example is with the correlation of transcriptomic and proteomic data [164]. The transcription of an mRNA may not necessarily indicate that the corresponding protein will also be produced. Thus, suggestions made about the active functional pathways based on transcriptomic profiling may not always be transferred to the proteomic level. Similarly, epigenomic changes occurring with chromosomes may modulate the expression of a gene assumed to be active from the genomics study. Thus, the integration of genomic and epigenomic data provides a more accurate picture of gene expression [165]. Transcriptomic data showing production of mRNA of a metabolic enzyme should be complemented by metabolomic studies to make unambiguous conclusions on the metabolic output. Of course, multi-omics studies should not always include all omics, since this may not be possible, and the rational and adequate choice of omics technologies is dependent on the nature of the research question.

The integration of complex omics datasets can provide information on molecular-level interconnections and mechanisms underlying different biological processes. A range of computational tools have been developed to handle the complexities of multi-omics integration which include similarity-based methods (NEMO and PINEPLUS), network-based approaches (WCGNA, Steiner, OmicsIntegrator, Weighted Multiplex Network, CONEXIC, and Joint NMF), correlation-based methods (MAPMan, CNAmet, PhenoLink, Paintomics, and IntegreOmics), Bayesian methods (TMD, MDI, PSDF, Joint BAYES, BCC, IBAG, MOFA), fusion-based methods (PFA and PSDF), and multivariate techniques (MixOmics) [166].

Furthermore, integration of classical omics studies (genomics, epigenomics, transcriptomic, proteomics, and metabolomics) with phenotypic analyses allows researchers to reproduce the entire pathway from genome to trait. Phenotypes include morphological, physiological, and biochemical characteristics such as plant height, root architecture, biomass, photosynthetic and respiratory efficiency, stress and disease tolerance, the composition of metabolites, hormones, and other biochemical substances that control growth and development [167]. Recent interdisciplinary advances in high-throughput phenotyping techniques have led to the emergence of a new discipline named “phenomics”. This field aims at collecting non-invasive imaging (i.e., RGB, chlorophyll fluorescence, hyperspectral and thermal imaging, and spectroscopy data) and developing comprehensive tools to integrate with other omics data [168,169]. These technologies in combination with Internet of Things (IoT) and blockchain devices can serve for collecting and analysing phenotypic data to provide information about plant responses to different conditions.

With regard to virus infections, it is worth noting that integrating metabolomics, genomics, transcriptomics, and proteomics allows researchers to elucidate complex mechanisms underlying plant–virus interactions, which allows formulation of critical insights into how specific gene expression correlates with metabolic reprogramming in virus-infected plants. For example, a study on maize plants infected with sugarcane mosaic virus (SCMV) showed that development of mosaic symptoms in SCMV-infected plants requires light illumination and is correlated with mitochondrial reactive oxidative species (mROS) accumulation. The transcriptomic and metabolomic analyses clearly demonstrated that SCMV infection elevates the enzymatic activity of pyruvate orthophosphate dikinase under light, resulting in malate overproduction and subsequent mROS accumulation which in turn contribute to the manifestation of light-dependent mosaic symptoms via mROS [170]. Similarly, metabolomic and transcriptomic profiling revealed that grapevine red blotch virus (GRBV) inhibits the phenylpropanoid pathway and its derivatives, decreasing the capacity of grapevine plant to produce antiviral compounds [78]. Conversely, other studies show that some viruses such as tobacco mosaic virus in tomato plants or cucumber mosaic virus in squash [171] can modulate phenolic compound production to counteract infection [172]. For other examples, see the comprehensive review by Jiang et al. [20].

## 8. Future Directions

**Figure 2:**
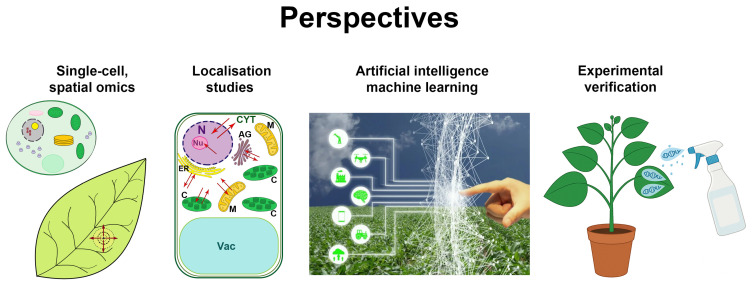
Future omics applications in the field of plant virology. Traditional omics or multi-omics studies have merely measured the average expression within a complex pool of cells and cell types which remain at distinct stages of infection and therefore should be complemented by single-cell and spatial (transcriptomics, epigenomics, proteomics and metabolomics) omics analyses. The complexity and scale of omics data requires additional analytical tools such as artificial intelligence (AI) and machine learning (ML). Taking into consideration that many biological processes are regulated at the intracellular localisation/compartmentalisation level, some innovative high-throughput super-resolution localisation techniques coupled with AI-based predictive models (protein/RNA localisomics, retainomics, sequestromics) should open up new additional opportunities in the application of current set of omics/multi-omics technologies in plant virology. Genetic and molecular data obtained by omics technologies should be verified by functional analysis. In conjunction with phenomics, plant physiology, and functional genomics complement each other and would be extremely useful for such verification, but first, phenomics techniques should be adapted for analysis of plant–virus interactions. N, nucleus; Nu, nucleolus; C, chloroplast; M, mitochondrion; AG, Golgi apparatus; CYT, cytosol; Vac, vacuole; ER, endoplasmic reticulum. Arrows indicate directions of virus movement from initially infected cells as shown in the “single-cell, spatial omics” column (see bottom left) or possible routes of macromolecular and metabolite trafficking (as shown in the “localisation studies” column).

As discussed above, both individual omics and multi-omics approaches have been used in plant virology to explore molecular mechanisms underlying susceptibility and resistance of plants to viruses and to pave the way for more efforts to engineer virus resistance. However, in the plant–virus context these approaches appear to significantly lag behind the general level of research in this field even if multi-omics studies are applied. Some future omics applications in the field of plant virology should include single-cell and spatial omics analyses, high-throughput super-resolution localisation techniques and AI-based predictive modelling. Genetic and molecular data obtained by omics technologies should also be verified by experimental functional analysis (Figure 2).

### 8.1. Single-Cell and Spatial Omics

Traditional omics or multi-omics studies have been instrumental in representing the differential accumulation of gene products. However, these bulk analyses merely measure the average expression within a complex pool of cells and cell types, thus masking the differences in expression between individual cells. Recent developments of single-cell and spatial (transcriptomics, epigenomics, proteomics, and metabolomics) omics techniques have already brought research breakthroughs in plant sciences [173], revolutionising our understanding of the regulatory mechanisms in multiple cell and tissue types. These approaches should be especially important in plant–virus interactions due to the absence of synchronous virus infections in plants [174]. Indeed, to establish infection, viruses continuously spread from cell to cell and then systemically through the plant long-distance transport system, the phloem. Thus, all the infected cells in plants represent different stages of infection from early to late, which apparently trigger distinct responses. Therefore, only by applying single-cell or more specifically spatial analysis to different areas of advancing infection foci would it be possible to relate molecular events in small groups of cells to a sequence of virus-induced changes. Moreover, virus infections in plants establish translocation signalling pathways [175], such as for amplification and spread of secondary siRNA (maintaining RNAi-based antivirus response) or SAR both of which are mobile factors of plant antiviral immunity and hence are of prime significance for omics analyses in a spatial manner. Thus, single-cell and spatial omics techniques should be applied to investigate plant–virus interactions in more detail.

### 8.2. Artificial Intelligence and Machine Learning

The complexity and scale of omics data requires sophisticated analytical tools. Among them are (i) artificial intelligence (AI) which creates computer systems to perform tasks associated with human intelligence, such as learning, problem solving, and decision-making, and (ii) machine learning (ML), a subset of AI, which concentrates on developing algorithms and statistical models that enable computers to perform tasks without specific programming and a deep learning (DL), the latter being a part within ML which is involved in training artificial neural networks to mimic the human brain’s architecture. These tools have already demonstrated great synergism with omics technologies in integrating multi-omics data and predicting plant responses under different stress conditions based on omics data including resistance genes and fungal effectors (reviewed in [25,176]). Thus, these AI-assisted omics technologies offer unprecedented opportunities to address critical gaps in plant–virus interactions.

### 8.3. Experimental Verification

Unlike conventional quantitative phenotyping, integrative phenomics involves comprehensive analysis of a set of physiological characteristics and considers the underpinning mechanisms at different layers of the biological system. This approach is valuable for practical applications because quantitative features of complex crop phenotypes and their response to environmental conditions including biotic and abiotic stresses cannot be understood and predicted based solely on a single genetic response. Physiological and molecular data should be verified by functional analysis. In conjunction with phenomics, plant physiology and functional genomics complement each other and would be extremely useful for such verification, but first, phenomics techniques should be adapted for analysis of plant–virus interactions.

### 8.4. Localisation Studies: Relocalisation, Retention, Sequestration—New Omics?

Protein subcellular localization and its dynamic changes is critical for understanding cellular function, particularly under stress or disease conditions, where protein relocalisation can change cellular responses. “Proviral” host proteins highjacked by the virus may be redirected to subcellular compartments where they contribute to essential viral processes, such as forming sites of viral replication, RNA synthesis, or virus movement [177]. On the other hand, antiviral host proteins may be removed from their natural subcellular localisations. Intracellular redistribution of host proteins is typically facilitated by direct or indirect interactions with viral proteins or RNA. For example, in infected groundnut plants, the groundnut rosette virus ORF3 protein interacts with the major nucleolar protein fibrillarin and relocalises it from the nucleolus through Cajal bodies into the cytoplasm. This ORF3-mediated relocalisation of fibrillarin is required for the formation of viral ribonucleoprotein particles competent for virus movement [16]. In another example, the tobacco rattle virus (TRV) 16K protein interacts with and relocalises a structural protein of Cajal bodies, coilin, to the nucleolus. The retention of coilin in the nucleolus is accompanied by activation of the salicylic acid-responsive antiviral defence responses and also plant recovery from TRV disease [178]. Numerous other cases of subcellular relocalisation, retention or sequestration of host proteins induced by viral infections are well documented and discussed by Rodriguez-Peña et al. [177]. Moreover, some long non-coding RNAs (lncRNAs) may operate as decoy molecules that bind and sequester proteins thereby inhibiting their normal functions [179,180]. Predictions and understandings of how non-coding RNAs may compete with various nucleic acids for binding of functional proteins will elucidate how lncRNA-mediated protein sequestration regulates plant–virus interactions [179].

Similarly, cellular localization of mRNA is essential for regulation of translational processes. mRNA molecules are transported to their target compartments to exert their biological functions via directed transport, protection from degradation and passive diffusion before local entrapment [181]. In response to various stress conditions mRNA translation rates are usually falling, with mRNA molecules being trafficked to so called stress granules (SG) for storing until the cell has been released from stress. Another type of RNA-containing granule are processing bodies (PB), where mRNA decay may occur. It is well known that plant viruses require components of both SGs and PBs for RNA replication and translation, but the entire structure and organisation of viral RNA granules and their links with mRNA regulatory networks has remained largely unelucidated [182].

Thus, many biological processes are regulated at the intracellular localisation/compartmentalisation levels. That is why development of innovative super-resolution high-throughput localisation (imaging) techniques coupled with AI-based predictive models as novel omics approaches (protein/RNA localisomics, retainomics, sequestromics) will open up new opportunities in application of current set of omics/multi-omics technologies in plant virology.

### 8.5. Biological Pitfalls

- Some additional biological aspects should be considered when planning omics related research on plant–virus interactions.
- Effect of viral tropism. Plant viral tropism refers to the bias a virus has for infecting and replicating within specific cell types or tissues [183]. Certain viruses may preferentially invade reproductive organs, meristems, and seeds, overcoming antiviral barriers to establish persistent infections [183]. In contrast, other viruses may be strictly limited to specific tissues, e.g., the phloem, the plant′s vascular tissue responsible for transporting organic nutrients [184]. It is also well known that viral replication sites vary depending on the virus type. Most RNA viruses replicate in the cytoplasm, while many DNA viruses replicate in the nucleus [185]. Some viruses, regardless of their genome type, establish specialised compartments within the cell to facilitate their replication [19,185]. These compartments may concentrate viral proteins and RNA to spatially organise the replication events.
- Mixed infections. In the field, mixed infections consist of two or more plant viruses, which often culminate in very complex multifaceted interactions between the host and viruses which may differentially modulate plant responses to environmental conditions, virus loads, virus tissue tropisms and light period [186]. Mixed infections very commonly occur and therefore omics-based research should recognise the complexities of mixed infections so that important knowledge can be obtained for effective control of plant viruses.

The concerns noted above likely represent only a fraction of the obvious pitfalls in the omics profiling of plant–virus interactions. Recent advances in super-resolution microscopy, single-cell, and spatial omics and AI will definitely be quite beneficial to overcome these concerns and to expand abilities of researchers in designing experiments on the omics studies in plant virology.

## 9. Conclusions

The omics, AI and combinatorial approaches discussed above have to be rigorously and carefully applied to produce novel detailed information on the mechanisms of both compatible and incompatible plant–virus interactions operating at different stages of infection. This will lead to accurate identification, functional characterisation and intracellular localisations of host determinants (genes, proteins, short and long non-coding RNAs and metabolites) controlling virus resistance or susceptibility. These constitute a set of targets which may be modified via genome editing (CRISPR-Cas), spray-induced silencing (RNA interference) or application of biochemical inhibitors/agonists of aspects of the different pathways. Such treatments may block different phases of infection, ranging from the very initial stages (virus inoculation) to suppression of virus accumulation (RNA replication), mitigation of viral disease symptoms and even full recovery. Such multilayer protection approaches will contribute to developing a sustainable and versatile way to control viral diseases in plants.

## Figures and Tables

**Table 1:** Omics approaches applied to plant–virus interactions studies.

Virus/Genus (RNA or DNA)	Plant	Examples of Modulated Pathways	References
**Transcriptomics**			
TYLCV/*Begomovirus* (DNA)	Tomato	Gibberellin-mediated antiviral defence	[28]
RBSDV/*Reovirus* (RNA)	Rice	m6A methylation	[29]
RSV/*Tennuivirus* (RNA)	Rice	m6A methylation	[29]
CGMMV/Tobamovirus (RNA)	Watermelon	m6A methylation	[30]
PVY/Potyvirus (RNA)	Potato	Photosynthesis, carbohydrate synthesis	[31]
TYLCV/*Begomovirus* (DNA)	Tomato	Defence response, ubiquitination	[32]
PVY/Potyvirus (RNA)	Potato	Resistance, susceptibility, photosynthesis	[33]
RSV/*Tennuivirus* (RNA)	Rice	Photosynthesis, flowering, stress/defence responses	[34]
CaLCuV/*Begomovirus* (DNA)	*Arabidopsis thaliana*	Translation machinery	[35]
TuMV/*Potyvirus* (RNA)			
CMV/*Cucumovirus* (RNA)	*Arabidopsis halleri*	Plant–pathogen interaction	[36]
BrYV/*Polerovirus* (RNA)			
SVBV/*Caulimovirus* (DNA)	Strawberry	Pigment metabolism, plant–pathogen interactions	[37]
TMV/*Tobamovirus* (RNA)	Tobacco	Reprogramming auxin-regulated gene expression	[38]
CMV/*Cucumovirus* (RNA)	Tobacco	Photosynthesis and chlorophyll metabolism	[39]
CCYV/*Crinivirus* (RNA)	Cucumber	Phenylpropanoid synthesis, phenylalanine metabolism	[40]
MIMV/*Nucleorhabdovirus* (RNA)	Maize	Immune receptor signalling, RNA silencing, developmental processes	[41]
CBSV/*Ipomovirus* (RNA)	Cassava		[42]
TYLCV/*Begomovirus* (DNA)	Tomato	Long non-coding and circular RNAs	[43]
CMV/*Cucumovirus* (RNA)	*Capsicum annuum*	Response to stress, defence response	[44]
BCTV/*Geminivirus* (DNA)	Sugar beet	Primary metabolic processes, volatile compounds	[45]
SLCCNV/*Begomovirus* (DNA)	Zucchini	Photosynthesis, plant–pathogen interactions	[46]
PVY/Potyvirus (RNA)	Potato	Poly(ADP)-ribosylation, methionine cycle	[47]
**Proteomics**			
CWMV/Furovirus (RNA)	*Nicotiana benthamiana*	Abscisic acid-mediated antiviral defence	[48]
ORMV/*Tobamovirus* (RNA)	*N. benthamiana*	Jasmonic and abscisic acid signalling, intracellular	[49]
MNSV/*Gammacarmovirus* (RNA)	Melon	Processes controlling redox balance and cell death	[50]
RSV/*Tennuivirus* (RNA)	Rice	Chlorophyll biosynthesis and cell death processes	[51]
TMV/*Tobamovirus* (RNA)	Tobacco	Photosynthesis	[52]
TYLCCNV/*Begomovirus* (DNA)	Tobacco	Stress defence, energy production, photosynthesis	[53]
CSMV/*Comovirus* (RNA)	*Vigna unguiculata*	Redox homeostasis, protein synthesis, defence, stress	[54]
PSbMV/*Potyvirus* (RNA)	*Pisum sativum*	Plant–pathogen response, lipid metabolism	[55]
SRBSDV/Fijivirus (RNA)	Rice	Defence superoxide dismutase and catalase activities	[56]
CPSMV/*Comovirus* (RNA)	*V. unguiculata*	Photosynthesis, stress response, and oxidative burst	[57]
TSWV/*Orthotospovirus* (RNA)	Tobacco	Cell death, host defence, metabolism	[58]
PVY/*Potyvirus* (RNA)	Potato	Methionine cycle	[59]
**Metabolomics**			
TRV/*Tobravirus* (RNA)	*A*. *thaliana*	Lipid and fatty acid metabolism	[60]
RBSDV/*Reovirus* (RNA)	Maize	Lipid and fatty acid metabolism	[61]
CCYV/*Crinivirus* (RNA)	Cucumber	Lipid metabolism	[62]
SCMV/*Potyvirus* (RNA)	Maize	Phenylpropanoid pathway	[63]
GLRaV-3/*Ampelovirus* (RNA)	Grapevine	Phenylalanine metabolism, salicylic acid pathway	[64]
HpMV/*Carlavirus* (RNA)			
ApMV/*Ilarvirus* (RNA)	Hop	Accumulation of monoterpenes hydrocarbons	[65]
HLVd (viroid)			
CMV/*Cucumovirus* (RNA)	Passion fruit	Levels of secondary metabolites and antioxidants	[66]
SaLV/*Potyvirus* (RNA)	Saffron	Composition of picrocrocin, safranal, and kaempferols	[67]
HgYMV/*Begomovirus* (DNA)	Horsegram	Accumulation of sugars, alkanes, and carboxylic acids	[68]
**Transcriptomics and** **Proteomics**			
MYMIV/*Begomovirus* (DNA)	Soybean	Cell cycle, cell-wall biogenesis, hormone assimilation	[69]
PVY/Potyvirus (RNA)	Potato	Plant immunity regulation	[70,71]
SCMV/*Potyvirus* (RNA)	Sugarcane	Photosynthesis	[72]
SCMV/*Potyvirus* (RNA)	Sugarcane	Sugar metabolism	[73]
TuMV/*Potyvirus* (RNA)	Cabbage	Calcium signalling pathways, heat shock responses	[74]
GFLV/Nepovirus (RNA)	*N. benthamiana*	Chitinase activity and hypersensitive response	[75]
**Transcriptomics and** **Metabolomics**			
PVY/Potyvirus (RNA)	Potato	Phenylpropanoids and antioxidant pathways	[76]
GRBaV/Grablovirus (DNA)	Grapevine	Abscisic acid, ethylene, and auxin pathways	[77]
GRBaV/Grablovirus (DNA)	Grapevine	Auxin-mediated pathways and photosynthesis	[78]
TuMV/Potyvirus (RNA)	*Brassica rapa*	Volatile organic compounds	[79]
TSWV/*Orthotospovirus* (RNA)	Tomato	Plant hormone signalling and flavonoid pathway	[80]
**Proteomics and** **Metabolomics**			
SLCMV/*Begomovirus* (DNA)	Cassava	Plant–pathogen interaction, hormone signalling	[81]
**Interactomics**			
TSWV/*Orthotospovirus* (RNA)	*N. benthamiana*	TSWV NSs-interacting proteins	[82]

TYLCV, tomato yellow leaf curl virus; RBSDV, rice black-streaked dwarf virus; RSV, rice stripe virus; CGMMV, cucumber green mottle mosaic virus; PVY, potato virus Y; CaLCuV, cabbage leaf curl virus; TuMV, turnip mosaic virus; CMV, cucumber mosaic virus; BrYV, Brassica yellows virus; SVBV, strawberry vein banding virus; TMV, tobacco mosaic virus; CCYV, cucurbit chlorotic yellows virus; MIMV, maize Iranian mosaic virus; CBSV, cassava brown streak virus; BCTV, beet curly top virus; SLCCNV, squash leaf curl China virus; CWMV, Chinese wheat mosaic virus; ORMV, oilseed rape mosaic virus; MNSV, melon necrotic spot virus; TYLCCNV, tomato yellow leaf curl China virus; CPSMV, cowpea severe mosaic virus; PSbMV, pea seed-borne mosaic virus; SRBSDV, southern rice black-streaked dwarf virus; TSWV, tomato spotted wilt virus; TRV, tobacco rattle virus; RBSDV, rice black-streaked dwarf virus; SCMV, sugarcane mosaic virus; GLRaV-3, grapevine leafroll-associated virus 3; HpMV, hop mosaic virus; ApMV, apple mosaic virus; HLVd, hop latent viroid; SaLV, saffron latent virus; HgYMV, horse gram yellow mosaic virus; MYMIV, mungbean yellow mosaic virus; GFLV, grapevine fanleaf virus; GRBaV, grapevine red blotch-associated virus; SLCMV, Sri Lankan cassava mosaic virus.

## Data Availability

Not applicable.

## Abbreviations

The following abbreviations are used in this manuscript:

PAMP	Pathogen-associated molecular pattern
PTI	PAMP-triggered immunity
ETI	Effector-triggered immunity
PRRs	Pattern recognition receptors
ROS	Reactive oxygen species
SA	Salicylic acid
SAR	Systemic acquired resistance
RNAi	RNA interference
TGS	Transcriptional gene silencing
PTGS	Post-transcriptional gene silencing
siRNAs	Small interfering RNAs
NGS	Next generation sequencing
RNAseq	RNA sequencing
MS	Mass spectrometry
NMR	Nuclear magnetic resonance
SNPs	Single nucleotide polymorphisms
GWAS	Genome-wide association studies
lncRNAs	Long non-coding RNAs
smRNAs	Small RNAs
DEGs	Differentially expressed genes
vsiRNAs	Viral small interfering RNAs
MTC	Methionine cycle
PARylation	Poly ADP-ribosylation
mROS	Mitochondrial reactive oxidative species
AI	Artificial intelligence
ML	Machine learning
SG	Stress granules
PB	Processing bodies
